# Transcriptome Analysis Reveals Novel Inflammatory Signalings to *Glaesserella parasuis* Infection

**DOI:** 10.3390/genes15081094

**Published:** 2024-08-20

**Authors:** Jingwen Lei, Xuexue Chen, Huanhuan Zhou, Zekai Zhang, Zhong Xu, Ke Xu, Hongbo Chen

**Affiliations:** 1 Laboratory of Genetic Breeding, Reproduction and Precision Livestock Farming, School of Animal Science and Nutritional Engineering, Wuhan Polytechnic University, Wuhan 430023, China; coralineee@163.com (J.L.); chenxx_1103@163.com (X.C.); hhzhou@whpu.edu.cn (H.Z.); zk.zhang@naosaisi.com (Z.Z.); 2 Hubei Provincial Center of Technology Innovation for Domestic Animal Breeding, Wuhan Polytechnic University, Wuhan 430023, China; 3 Key Laboratory of Animal Embryo Engineering and Molecular Breeding of Hubei Province, Wuhan 430023, China; xz8907@163.com; 4 Hubei Hongshan Laboratory (HHL), Wuhan 430070, China

**Keywords:** *Glaesserella parasuis*, transcriptome sequencing, systemic inflammatory response, cell response, disease-resistance breeding

## Abstract

*Glaesserella parasuis* (GPS) can cause severe systemic inflammation in pigs, resulting in huge economic losses to the pig industry. At present, no effective method is available for the prevention and control of GPS infection. Molecular breeding for disease resistance is imminent, but disease-resistance genes have not been identified. To study the mechanism of systemic acute inflammation caused by GPS, we established three in vitro infection models (3D4/21 cells, PK15 cells, and PAVEC cells) according to its infection path. There was no significant difference in apoptosis among the three kinds of cells after 12 h of continuous GPS stimulation, while inflammatory factors were significantly upregulated. Subsequent transcriptome analysis revealed 1969, 1207, and 3564 differentially expressed genes (DEGs) in 3D4/21 cells, PK15 cells, and PAVEC cells, respectively, after GPS infection. Many of the DEGs were predicted to be associated with inflammatory responses (C3, CD44, etc.); cell proliferation, growth and apoptosis; gene expression; and protein phosphorylation. Key signaling pathways, including S100 family signaling, bacteria and virus recognition, and pathogen-induced cytokine storm signaling, were enriched based on Ingenuity Pathway Analysis (IPA). Furthermore, a total of three putative transmembrane receptors and two putative G-protein-coupled receptors, namely F3, ICAM1, PLAUR, ACKR3, and GPRC5A, were identified by IPA among the three types of cells. ACKR3 and GPRC5A play pivotal roles in bacterial adhesion, invasion, host immune response and inflammatory response through the S100 family signaling pathway. Our findings provide new insights into the pathological mechanisms underlying systemic inflammation caused by GPS infection in pigs, and they lay a foundation for further research on disease-resistance breeding to GPS.

## 1. Introduction

Pork is one of the most important sources of meat. Health has become one determinant of productivity, profitability, and animal welfare in pig production [1]. Glaesserella parasuis (GPS) is an important bacterial causative agent of Glässer’s disease in pigs. This disease has a variety of pathological features, including fibrinous polyserositis, arthritis, pleurisy, and meningitis [2], and it causes severe post-weaning morbidity and mortality in pig herds worldwide [2,3], resulting in significant economic losses.

Specifically, GPS invades the host through the respiratory tract, wherein it expresses IgA protease during the colonization of the upper respiratory tract, thus evades host mucosal immunity via destroying the IgA heavy chain structure [3]. Breaking through the pulmonary defense has been recognized as the other major way for GPS systematic diffusion [4], in which the immunity of porcine alveolar macrophages, the first line of pulmonary defense, could be weakened [5]. Then, the bacterium will cause blood vessel damage and sepsis once it invades into the bloodstream [6]. Eventually, it will spread throughout the body, such as the kidneys and brain, leading to intense inflammations and even the death of the host [7].

Therefore, the control and prevention of GPS infection are urgently needed [8]. At present, there are many disadvantages in the prevention and control of GPS infection, such as a high dependence on antibiotics and inadequate protection of vaccines [9], which affects the sustainable development of the pig industry. It is essential to propose alternative strategies that can prevent GPS infection efficiently. Breeding disease-resistant pigs through molecular breeding has been considered as an effective alternative [9]. Achieving molecular breeding through gene editing is feasible and valuable in improving livestock traits; for example, the CD163 receptor knockout pigs will have complete resistance to the highly pathogenic porcine reproductive and respiratory syndrome virus (PRRSV) [10].

The key of disease-resistance breeding lies in mining porcine disease-resistant genes. So far, studies on the pathogenesis of GPS mainly focus on bacterial immune escape and virulence factors, and the mechanisms related to inflammatory response and inflammatory injury that play an important role in the pathogenesis of Glässer’s disease are still unclear [11]. If we can fully understand the mechanism of systemic acute inflammation caused by GPS infection and carry out molecular breeding, then we can increase the possibility of preventing and successfully treating GPS.

Therefore, in this study, three different in vitro GPS infection models were established under the same challenge conditions and subjected to transcriptome analysis. Specifically, we fully considered the in vivo invasion pathway of the bacteria and selected porcine alveolar macrophages 3D4/21, porcine aortic vascular endothelial cells (PAVECs) and porcine renal epithelial cells (PK-15) as the study models, which are important models for studying immune escape, tissue inflammation and the vascular injury of GPS infection, respectively [12,13,14]. We then screened for common biological processes and inflammation-related genes and pathways in three comparisons. Our findings will improve our understanding of the mechanism underlying the systemic inflammatory response induced by GPS while providing guidance for the control and prevention of GPS infection.

## 2. Materials and Methods

### 2.1. Bacterial Strain Culture

The GPS SH0165 strain, a highly virulent strain of serovar 5, was used in this study. It was cultured in tryptic soy broth (Difco Laboratories, BD, Radnor Township, PA, USA) supplemented with 0.01% nicotinamide adenine dinucleotide (Sigma-Aldrich^®^, Taufkirchen, Germany) and 10% fetal bovine serum (Gibco, NY, USA) under 37 °C. The selected single colony was cultured overnight in tryptic soy broth at 37 °C and 225 rpm until OD_600_ reached approximately 0.7.

### 2.2. Cell Culture

The 3D4/21 cell line and PK15 cell line were provided by Dr. Zhao from Huazhong Agricultural University [15,16]. PAVEC cells were previously successfully isolated and identified in our laboratory [17]. The 3D4/21 cells were cultured in complete growth media containing RPMI 1640 medium (Gibco, USA) supplemented with 10% fetal bovine serum (Gibco, NY, USA). The PAVEC cells were cultured in complete growth media containing M-199 medium (Gibco, NY, USA) containing 10% fetal bovine serum (Gibco, NY, USA). The PK15 cells were cultured in complete growth media containing Dulbecco’s Modified Eagle Medium (DMEM)/high-glucose medium (HyClone, UT, USA) containing 10% fetal bovine serum (Gibco, NY, USA). All kinds of cells were cultured in a humidified cell incubator at 37 °C and 5% CO_2_.

### 2.3. Cell Infection

3D4/21 cells, PAVEC cells, and PK15 cells were seeded into 12-well plates and infected with GPS SH0165 strain with a multiplicity of infection (MOI) of 100. After co-culture for 12 h at 37 °C with 5% CO_2_, the supernatant was removed. The cells were collected and washed three times with 1% sterile phosphate-buffered saline for transcriptomic analysis. Each control cell line was infected with its own culture medium and treated in the same way. There were four separate replicates for 3D4/21 cells and three separate replicates for PAVEC and PK15 cells in each group.

### 2.4. Detection of Apoptosis

Apoptosis was analyzed using a Calcein-AM/PI kit (Beyotime, Shanghai, China). After 12 h of bacterial infection, the cells were treated according to the manufacturer’s protocol. The samples were analyzed using an inverted fluorescence microscope (Olympus Inc., Tokyo, Japan) and fluorescent enzyme labeling instrument (Molecular Devices, SJ, USA). Four individual replicates were utilized in these experiments.

### 2.5. Cytokines Analysis

Cytokines expression at the mRNA level was carried out with the qRT-PCR method. Total RNA was extracted from cells with Trizol reagent (Tiangen, Beijing, China) and then reverse-transcribed to cDNA using reverse transcriptase (TaKaRa, Osaka, Japan). The cDNA was amplified and measured using TB Green^®^ Premix Ex TaqTM (TaKaRa, Osaka, Japan) on a QuantStudio 6 Flex Real-time PCR System (Thermo Fisher Scientific, Waltham, USA). The reaction conditions were 95 °C for 30 s; followed by 40 cycles of 95 °C for 10 s, 60 °C for 30 s; and ended with a melting curve analysis. The 2^−ΔΔCt^ method was used to calculate the relative expression levels between the treatment and control groups. The reference gene RPL32 was used for data normalization in mRNA analysis. The primer sequences for inflammatory cytokines are listed in Supplementary Table S1. The bar chart was made using Graphpad Prism 9.

### 2.6. Transcriptome Sequencing and Differentially Expressed Gene (DEG) Analysis

RNA was extracted from cells and sent to the Genergy Biotechnology Corporation (Shanghai, China) for mRNA purification, library preparation and sequencing. A total of 20 samples (three replicates for PAVEC and PK15 cells in each group, four replicates for 3D4/21 cells in each group) were sequenced on the Illumina Hiseq 2500 platform. Raw sequencing data were filtered and quality controlled to obtain clean reads. These reads were mapped to the pig reference genome (*Sscrofa11.1*) using HISAT2 [18]. Then, gene fragments were estimated using Stringtie [19]. Expression levels for mRNAs were performed by calculating FPKMs (fragments per kilobase of exon model per million reads mapped). Next, differentially expressed genes (DEGs) were analyzed using the DESeq2 R package. Genes with a *p*-value < 0.05 and |log_2_ fold change| > 1 were considered differentially expressed. The associated volcano maps and Venn maps were produced using R packages. We used Ingenuity Pathway Analysis (IPA) (Ingenuity Systems, Qiagen, California, USA) software to perform molecular functional analysis of the differential expression gene code. To estimate the reliability of RNA-seq results, six DEGs were randomly selected and validated by qRT-PCR. The primer sequences for transcriptome validation are listed in Supplementary Table S1.

### 2.7. Enrichment Analysis of DEGs

Gene Ontology (GO) enrichment analysis was performed using the Database for Annotation, Visualization and Integrated Discovery (DAVID) website (https://david.ncifcrf.gov/, accessed on 27 June 2021). DEGs were uploaded to IPA software for canonical pathway analysis, network discovery, etc. For canonical pathway analysis, disease, and function, a z-score > 2 was defined as the significant activation threshold, and a z-score < −2 was defined as the significant inhibition threshold. All terms with a *p*-value < 0.05 were considered significantly enriched by DEGs. Putative receptor-associated network maps were made using IPA software, and putative receptor-related Sankey plots were made using Origin 7.

### 2.8. Statistical Analysis

Results were presented as mean ± standard deviation (SD). The statistical significance between the control and treated groups was analyzed with Student’s t-test in GraphPad Prism 9. *p*-value < 0.05 was considered statistically significant. * *p* < 0.05; ** *p* < 0.01; and ns = not significant.

## 3. Results

### 3.1. Constructions of the In Vitro GPS Infection Models

**Figure 1:**
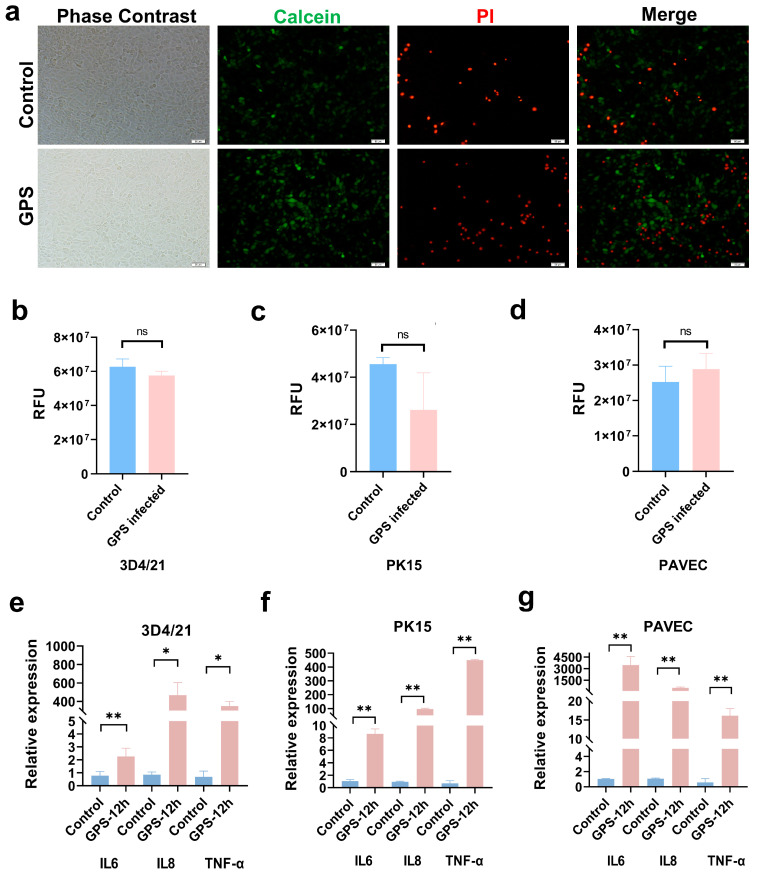
Constructions and verification of the in vitro GPS infection model. (**a**) The fluorescence (lower) and phase contrast microscopy images of 3D4/21 cells in the experimental and control groups. The green, fluorescent marks were Calcein-stained live cells, and the red fluorescent marks were PI-stained dead cells. (**b**) The difference in the relative fluorescence (RFU) values of 3D4/21 cell apoptosis between the experimental and control groups. (**c**) The difference in the RFU values of PK15 cell apoptosis between the experimental and control groups. (**d**) The difference in the RFU values of PAVEC cell apoptosis between the experimental and control groups. (**e**) Analysis of the difference in the expression levels of inflammatory cytokines in 3D4/21 cells between the experimental and control groups. (**f**) Analysis of the difference in the expression levels of inflammatory cytokines in PK15 cells between the experimental and control groups. (**g**) Analysis of the difference in the expression levels of inflammatory cytokines in PAVEC cells between the experimental and control groups. * *p* < 0.05; ** *p* < 0.01; and ns = not significant.

In our previous study, 3D4/21 cells and GPS (MOI 100:1) were co-cultured in a humidified cell incubator at 37 °C and 5% CO_2_ for 6 h, and the bacterial adhesion assays were conducted after cleaning. The number of bacteria that adhered to each 3D4/21 cell was approximately one per cell [20]. Compared with control cells, there was no significant difference in the incidence of apoptosis of 3D4/21 cells after 12 h of continuous GPS stimulation (Figure 1a). The apoptosis of 3D4/21 cells was not significant until 12 h following GPS infection (Figure 1b). After infecting 3D4/21 with GPS bacteria, a large zone of cell death was evident at 48 h, but no significant change was evident in the cell phenotype in the first 8 h (Figure S1).

Consistent with 3D4/21 cells, there was no significant difference in the incidence of apoptosis between the experimental group and the control group in PK15 and PAVEC cells (Figure 1c,d). The expression of the GPS-induced inflammatory response was detected by qRT-PCR. As shown in Figure 1f,g, the mRNA levels of some inflammatory factors (IL6, IL8, and TNF-α) were significantly upregulated in the treatment group compared with the control group in three cell lines (*p* < 0.05). Previous studies of GPS-challenged 3D4/21 and PAVEC cells have also proved that 12 h is the promising time point [13,21]. Thus, we chose 12 h as the ideal time point and conducted follow-up transcriptome analysis.

### 3.2. Differentially Expressed Genes (DEGs) after GPS Infection

RNA-Seq was used to detect the mRNA expression profiles of GPS-infected cells and control cells. A total of 20 RNA libraries were constructed with the total RNA extracted from the following groups: the 3D4/21_WT group, the 3D4/21_GPS group (four replicates in each group), the PK15_WT group, the PK15_GPS group, the PAVEC_WT group, and the PAVEC_GPS group (three replicates in each group). Approximately 950 million clean reads were generated for all of the libraries, and more than 95% of the clean reads per sample could be mapped to the pig reference genome (*Sscrofa11.1*), suggesting a good sequence quality. Principal components analysis (PCA) was consistent among the group replicates (Figure S2).

**Figure 2:**
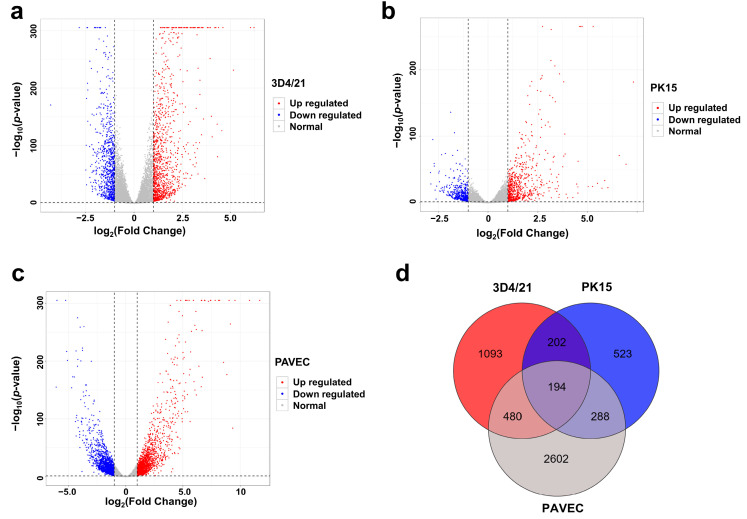
Differentially expressed genes (DEGs) analysis. (**a**) Volcano plot reveals significant DEGs in the comparison of 3D4/21_GPS vs. 3D4/21_WT. (**b**) Volcano plot reveals significant DEGs in the comparison of PK15_GPS vs. PK15_WT. (**c**) Volcano plot reveals significant DEGs in the comparison of PAVEC_GPS vs. PAVEC _WT. (**d**) A Venn diagram showing the DEGs identified from the three comparisons.

Upon comparison of the 3D4/21_WT cells, a total of 1969 significantly DEGs, including 1131 upregulated and 838 downregulated genes, were revealed in the 3D4/21_GPS samples (Figure 2a, Table S2). Differential gene expression analysis between PK15_GPS and PK15_WT showed 1207 significantly DEGs, including 692 upregulated genes and 515 downregulated genes (Figure 2b, Table S2). Differential gene expression analysis between PAVEC_GPS and PAVEC_WT showed 3564 significantly DEGs, including 1785 upregulated genes and 1779 downregulated genes (Figure 2c, Table S2). The Venn diagram in Figure 2d shows that 194 DEGs are shared among the three cells. IPA analysis also provides a reference for the molecular function of differential genes. The protein types corresponding to DEGs mainly include transmembrane receptors, G protein-coupled receptors, translation regulators, transcription factors, enzymes and kinases. Overall, 24, 19, and 28 putative transmembrane receptors were upregulated in the 3D4/21_GPS vs. 3D4/21_WT group, PK15_GPS vs. PK15_WT group, and PAVEC_GPS vs. PAVEC_WT group, respectively (Figure S3a, Table S3). In addition, a total of 20, 10, and 19 putative G-protein-coupled receptors were upregulated in the 3D4/21_GPS vs. 3D4/21_WT group, PK15_GPS vs. PK15_WT group, and PAVEC_GPS vs. PAVEC_WT group, respectively (Figure S3b, Table S4).

**Figure 3:**
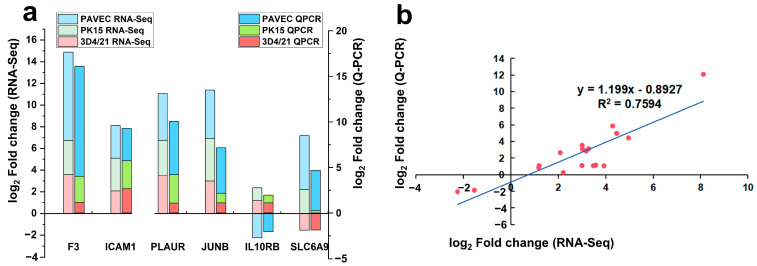
Validation of transcriptome analysis. (**a**) The qRT-PCR and RNA-seq assay results for common genes in three comparisons. (**b**) Correlation log2FC from the qRT-PCR and RNA-seq assays.

A total of six genes, including three receptor genes, were selected for transcriptome validation. The results showed a consistent upregulation tendency in both the qRT-PCR and RNA-seq results in three kinds of cells (Figure 3a). As shown in the Figure 3b, both methods displayed a strong correlation (R^2^ = 0.76).

### 3.3. Enrichment Analysis of DEGs

**Figure 4:**
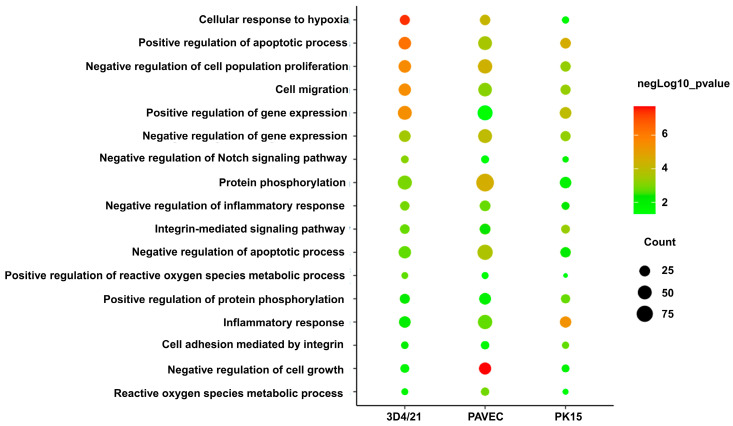
Common enriched gene ontology functional classifications of differential expression genes (DEGs) between GPS treatment group and control group.

The DEG functions were identified using the DAVID website. Overall, 219, 204, and 306 significantly enriched entries were identified in the biological process category in the 3D4/21_GPS vs. 3D4/21_WT groups, PK15_GPS vs. PK15_WT groups, and PAVEC_GPS vs. PAVEC _WT groups (Table S5). In all three comparisons, most of the enriched biological processes were significantly associated with inflammatory responses, cell proliferation, growth and apoptosis, gene expression, active oxygen metabolism, and protein phosphorylation (Figure 4). In the three comparisons, ten genes (*PXK*, *CSF1*, *RELB*, *C3*, *NFKB2*, *IL1A*, *NR4A1*, *REL*, *CD44*, and *EPHA2*) were involved in inflammatory response and the regulation of inflammatory response in the three comparisons.

**Figure 5:**
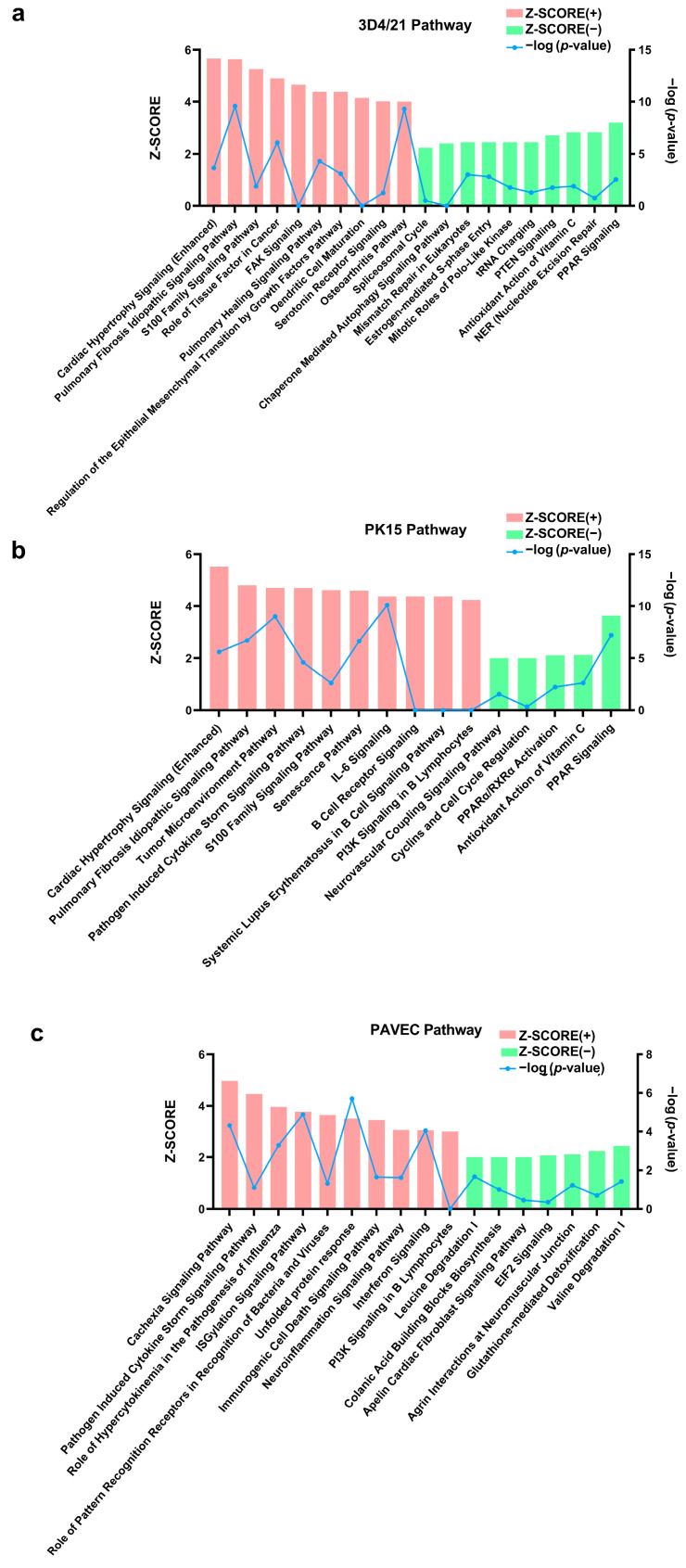
Top ten upregulated and downregulated pathways in three types of cells. If there are less than 10 items, all of them are displayed. Categories are shown in terms of the z-score, as represented by the left y-axis and the −log (*p*-value), represented by the right y-axis. (**a**) Top ten upregulated and top ten downregulated pathways in the comparison of 3D4/21_GPS vs. 3D4/21_WT. (**b**) Top ten upregulated and top ten downregulated pathways in the comparison of PK15_GPS vs. PK15_WT. (**c**) Top ten upregulated and top ten downregulated pathways in the comparison of PAVEC_GPS vs. PAVEC_WT.

Based on Ingenuity Pathway Analysis (IPA), 177 pathways were significantly enriched in the 3D4/21_GPS vs. 3D4/21_WT group, among which 16 pathways were downregulated (Table S6). As shown in Figure 5a, the pulmonary fibrosis idiopathic signaling pathway, pulmonary healing signaling pathway, cardiac hypertrophy signaling pathway, and S100 family signaling pathway were significantly upregulated. In contrast, signaling pathways, such as PPAR, were significantly downregulated. A total of 183 significant pathways were identified in PK15_GPS vs. PK15_WT. Among them, five signaling pathways, such as PPAR and PPARα/RXRα activation, were significantly downregulated, and the cardiac hypertrophy signaling, pulmonary fibrosis idiopathic signaling pathway, and S100 family signaling pathway were significantly upregulated (Table S6, Figure 5b). In the PAVEC_GPS vs. PAVEC_WT group, 67 significant pathways were identified, including the cachexia signaling pathway, the pathogen-induced cytokine storm signaling pathway, the role of pattern recognition receptors in the recognition of bacteria and viruses, the immunogenic cell death signaling pathway, and the neuroinflammation signaling pathway. Among them, seven signaling pathways, such as EIF2 signaling, were significantly downregulated (Table S6, Figure 5c). In terms of diseases and function analysis, 267, 313, and 266 items were enriched in each of the three cell types (Table S7). For example, the invasion of cells, activation of cells, and other functions were significantly activated (Figure S4a–c).

**Figure 6:**
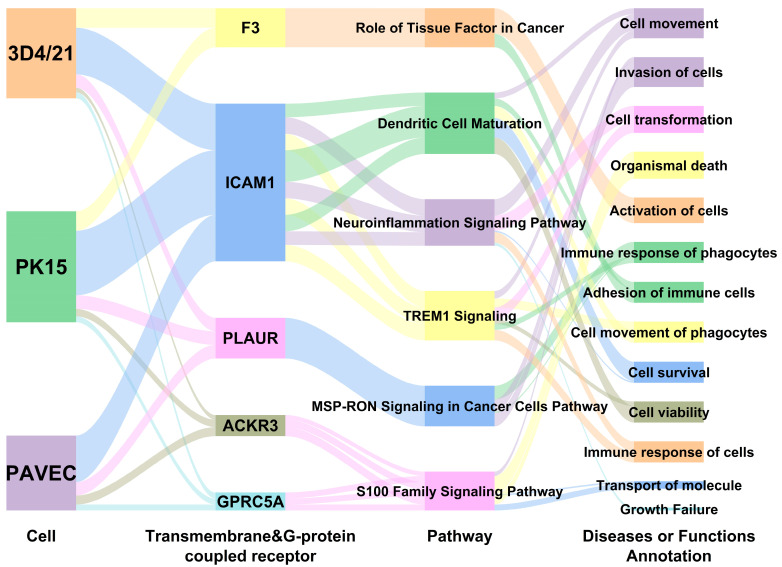
The Sankey diagram of putative common receptors by IPA in three comparisons.

For interaction networks, there were 25 interaction networks per analysis (Table S8). The network diagrams of putative common receptors participation are shown in Figure S5. Run-through analysis with consideration of cell types, putative receptors, signaling pathways, and functions further discovered a clear diagram of the biological implications for the five candidate receptors. As seen in Figure 6, roles of the five putative common receptors by IPA are involved in cell invasion, adhesion, activation, as well as molecular delivery, immune response, and organ death, implying the pivotal meanings in mediating GPS infection. In addition, ICAM1 was involved in three pathways in all three types of cells: the TREM1 signaling pathway, neuroinflammation signaling pathway, and dendritic cell maturation. ACKR3 and GPRC5A participate in the S100 family signaling pathway in three kinds of cells.

## 4. Discussion

Pigs are important agricultural animals and provide abundant meat products for humans [22]. GPS infection can elicit systemic acute inflammation and has been recognized as one of the major pathogens causing economic losses in the global swine industry [23,24]. Based on the practical needs of the industry, realizing the control and treatment of GPS through molecular breeding is necessary for disease resistance [9]. The key genes for disease-resistance breeding remain to be explored. Inflammatory immune response and inflammatory damage play an important role in the pathogenesis of Glässer’s disease, but the relevant mechanisms are still unclear [11].

The present research incorporated transcriptome sequencing to analyze the DEGs between GPS-infected and wild-type cells. The three comparisons showed that GPS infection of different cell types consistently affected biological processes, such as inflammatory responses, cell proliferation, growth and apoptosis, gene expression, active oxygen metabolism, and protein phosphorylation regardless of cell type. A series of genes, including *C3*, *PXK*, *CSF1*, *RELB*, *NFKB2*, *IL1A*, *NR4A1*, *REL*, *CD44* and *EPHA2*, were involved in inflammation and inflammation regulation in all three cell types. Among these genes, changes in the expression of acute-phase complement C3 and adhesion molecule CD44 have been previously reported in a study of GPS-infected pig spleens [25]. NR4A1 has been identified as an important regulator of immune and inflammatory responses [26], while the EphA2 gene has been shown to interfere with the response to infection in studies of host interactions with *Mycobacterium tuberculosis* [27].

At present, the receptors involved in GPS bacterial adhesion and invasion remains unclear. IPA analysis also gave us some clues about possible receptors. The common putative membrane receptors of the three cell types are F3, ICAM1, and PLAUR; the common putative G-protein-coupled receptors are ACKR3 and GPRC5A. Among these five shared receptors, F3, PLAUR, ICAM1, and ACKR3 also appeared in previous transcriptome analysis of GPS-infected 3D4/21 cells with different MOI challenges [21]. In studies of African swine fever (AFV) virus infection, the inflammation in infected porcine alveolar macrophages was significantly reduced upon inhibition of the F3 transcription [28]. Our study also found that F3 is closely associated with immune response, antimicrobial response, immune disease and inflammatory response in GPS infection. The upregulation of ICAM1 induced by E. coli increased blood–brain barrier breakdown and neuroinflammatory response [29]. ICAM1 regulates the movement of white blood cells, adhesion to blood vessels, and inflammation; in some viral infections, it has been recognized as the receptor to release virus RNA in the patient’s host cells, thus exacerbating the infection [30]. Our interaction network analysis revealed that ICAM1 is associated with GPS-infected immune diseases. ICAM1 functions primarily through the TREM1 signaling pathway. In studies of acute *Streptococcus suis* infection, TREM1 has immunomodulatory functions, which is protective to the occurrence and development of pathogenic effects [31].

The putative common G protein-coupled receptors, ACKR3 and GPRC5A, play a vital role in pathological inflammation [32,33]. ACKR3 is a core receptor candidate that facilitates the entry of HIV-1 and HIV-2 in vitro [34]. It has been increasingly implicated in neuroinflammation, and its induced disruption of the blood–brain barrier may be a potential co-pathological mechanism [35]. GPRC5A, a novel putative receptor discovered in this study, is essential in inflammation and immunity and has been recognized as a potential new target for inflammation and immunotherapy [33]. GPRC5A knockdown leads to NF-κB activation, which promotes lung inflammation [36]. The only signaling pathway in which the two putative G protein-coupled receptors are involved in all three types of cells is the S100 signaling pathway. The S100 protein family plays a vital role in host immune responses to a variety of diseases and is involved in both intracellular and extracellular processes, including apoptosis, migration, protein phosphorylation, differentiation, proliferation, and inflammation [37]. The S100 calcium-binding protein A4 (S100A4) and S100 calcium-binding protein A6 (S100A6) genes were significantly upregulated in the lungs, spleen, and lymph nodes of GPS-infected pigs and GPS-infected PK15 cells [14]. Porcine S100 calcium-binding protein A8 (S100A8) and S100 calcium-binding protein A9 (S100A9) have been shown to be molecular signatures of GPS responses [38]. In addition, the S100 calcium-binding protein A12 (S100A12) gene is abundant in the immune tissue of pigs and is significantly upregulated when infected with GPS [39].

Of the three cell types, 3D4/21, or macrophages, play an essential role as the effector of the immune response and contribute the first line of pulmonary defense against invading pathogens [14,40]. As the study points out, macrophages are highly heterogeneous and plastic when faced with pathogens [41]. In our results, both classical and alternative activation occurred in macrophages after GPS infection (because the z-score of classical activation was 1.961, it was not shown in Table S6). Classical and alternative activation pathways are related to the M1 and M2 polarization of macrophages, respectively. These results indicate that macrophages have a high degree of heterogeneity and plasticity when facing GPS, which is worthy of further study.

This study also has some areas that can be improved. For example, a promised negative control would be infection with the same serotype of the modified GPS strain that is not virulent. But this will be challenging work, since the landscape of the virulent factors for GPS has not been fully discovered. In addition, conducting the same test on cell lines originating from the porcine respiratory tract might provide more information. In future studies, we will take these two points into account to provide more definitive insights.

## 5. Conclusions

In this study, we constructed three types of in vitro GPS infection models and analyzed the response of different cells to GPS. No significant difference in apoptosis was evident after 12 h of continuous GPS stimulation in any of the three cell types, whereas inflammatory factors were significantly upregulated. Subsequent transcriptome analysis revealed that many genes (*C3*, *CD44, ACKR3*, *GPRC5A*, etc.) and the S100 signaling pathway were involved in the inflammatory response. The activation of the S100 signaling pathway, along with the upregulation of candidate receptors, ACKR3 and GPRC5A, are closely associated with acute inflammation caused by GPS infection. Our findings provide new insights into the pathological mechanisms underlying systemic inflammation induced caused by GPS infection in pigs.

## Data Availability

Raw data can be obtained by contacting the corresponding author.

## Supplementary Materials

The following supporting information can be downloaded at https://www.mdpi.com/article/10.3390/genes15081094/s1, Figure S1: GPS-induced epigenetic changes in 3D4/21 cells; Figure S2: Principal component analysis for all cells; Figure S3: The landscape of putative receptors that are differentially regulated in three GPS challenged cellular models; Figure S4: Top ten upregulated and top ten downregulated functions in three types of cells; Figure S5: Networks for putative receptor candidates in different kinds of cells. Table S1: Primes list; Table S2: The differentially expressed genes in three comparisons; Table S3: Transmembrane receptors inferred by IPA software list; Table S4: G-protein-coupled receptors inferred by IPA software list; Table S5: Biological process categories in three comparisons; Table S6: Canonical pathways in three comparisons; Table S7: Diseases and functions in three comparisons; Table S8: Networks in three comparisons.
