# Supplementary material for: Transcriptome Analysis Reveals Novel Inflammatory Signalings to Glaesserella parasuis Infection
Source: Genes (Basel). 2024 Aug 20;15(8):1094. doi: 10.3390/genes15081094 (PMC11353251; doi:10.3390/genes15081094)
Supplement: Supplementary file 1 [file genes-15-01094-s001.zip › Supplementary figures.pdf]

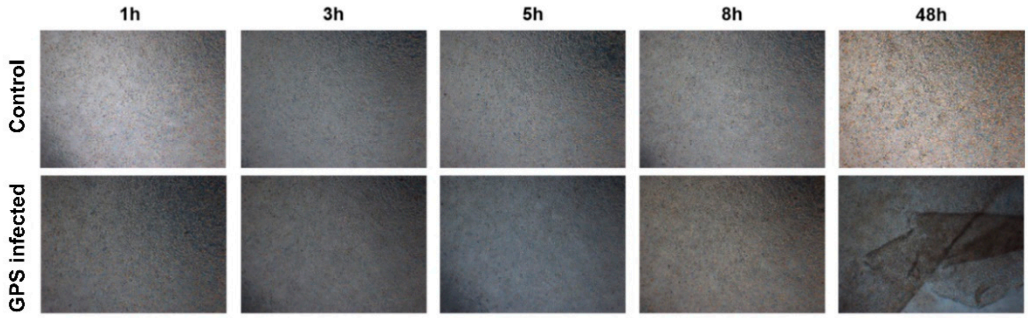


**Figure S1.** Gps-induced epigenetic changes in 3D4/21 cells.

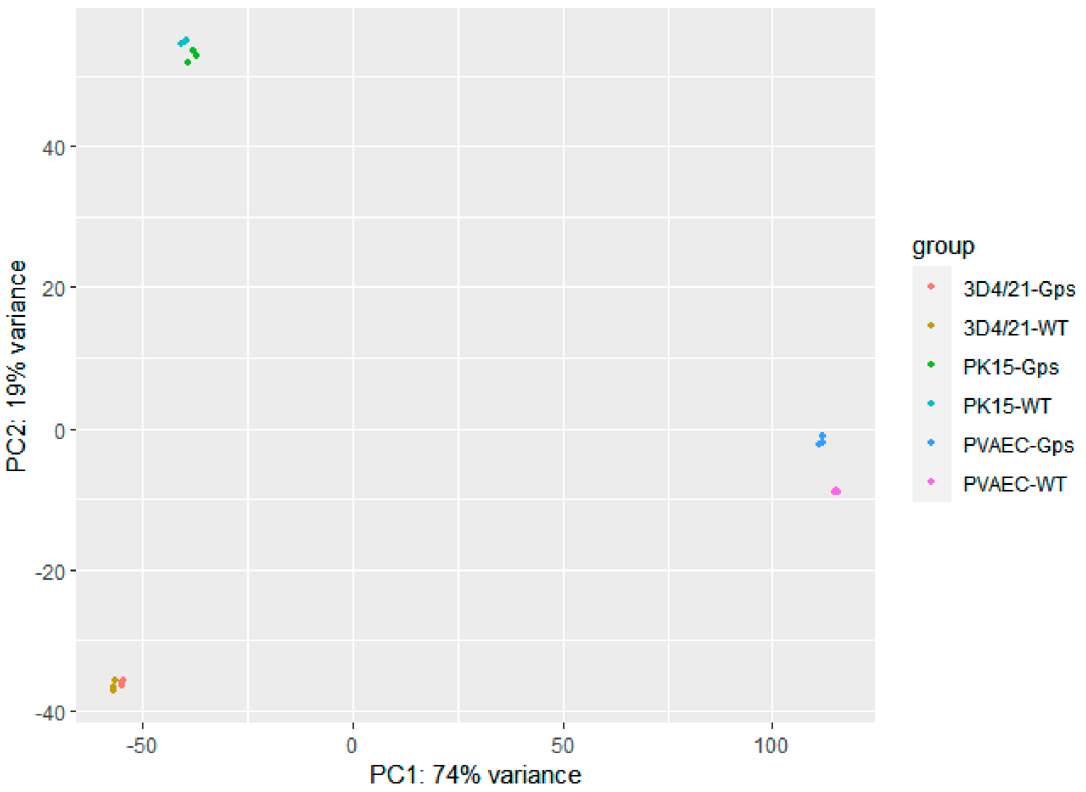


**Figure S2.** Principal component analysis for all cells. Principal component 1 (PC1) and principal component 2 (PC2) were identified by logarithm transformation in DESeq2.

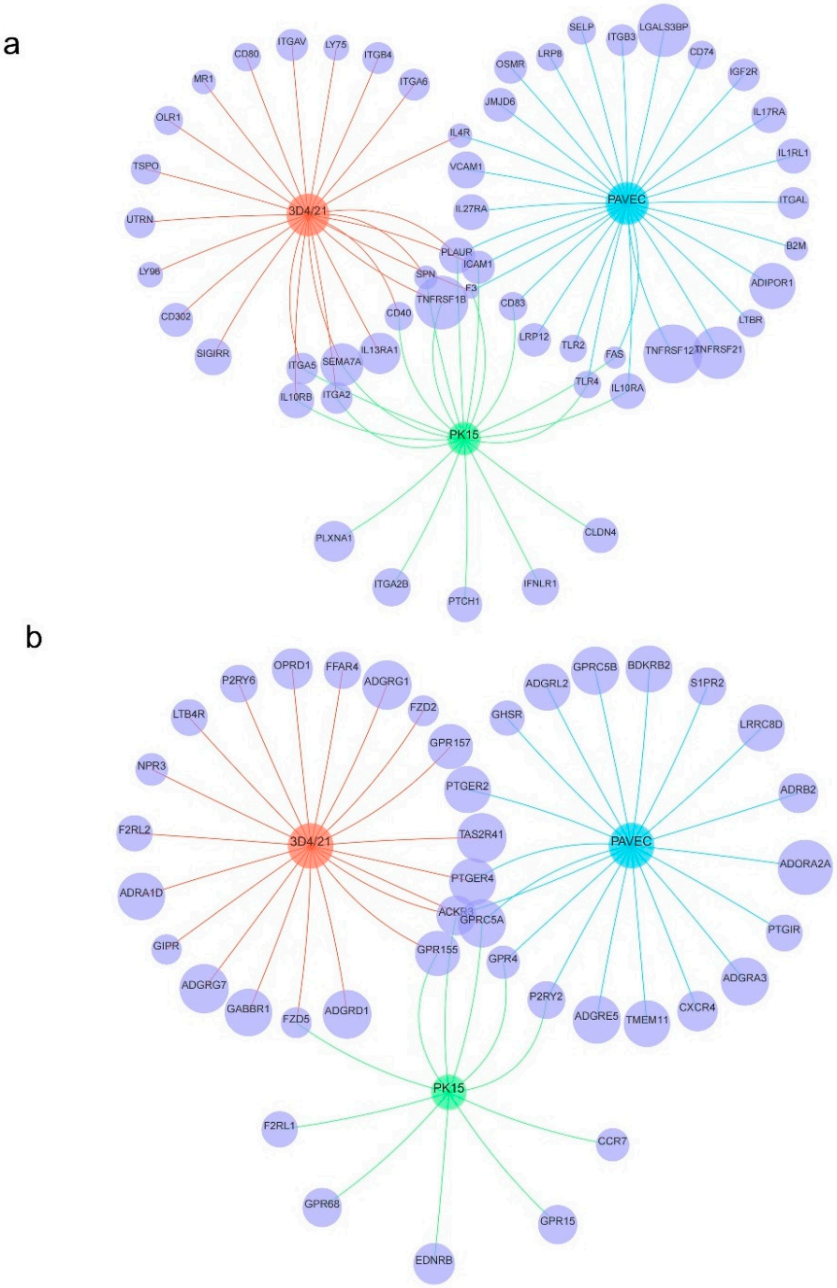


Figure S3. The landscape of putative receptors that are differentially regulated in three GPS challenged cellular models. (a) Network of G-protein coupled receptors in three comparisons. (b) Network of transmembrane receptors in three comparisons.

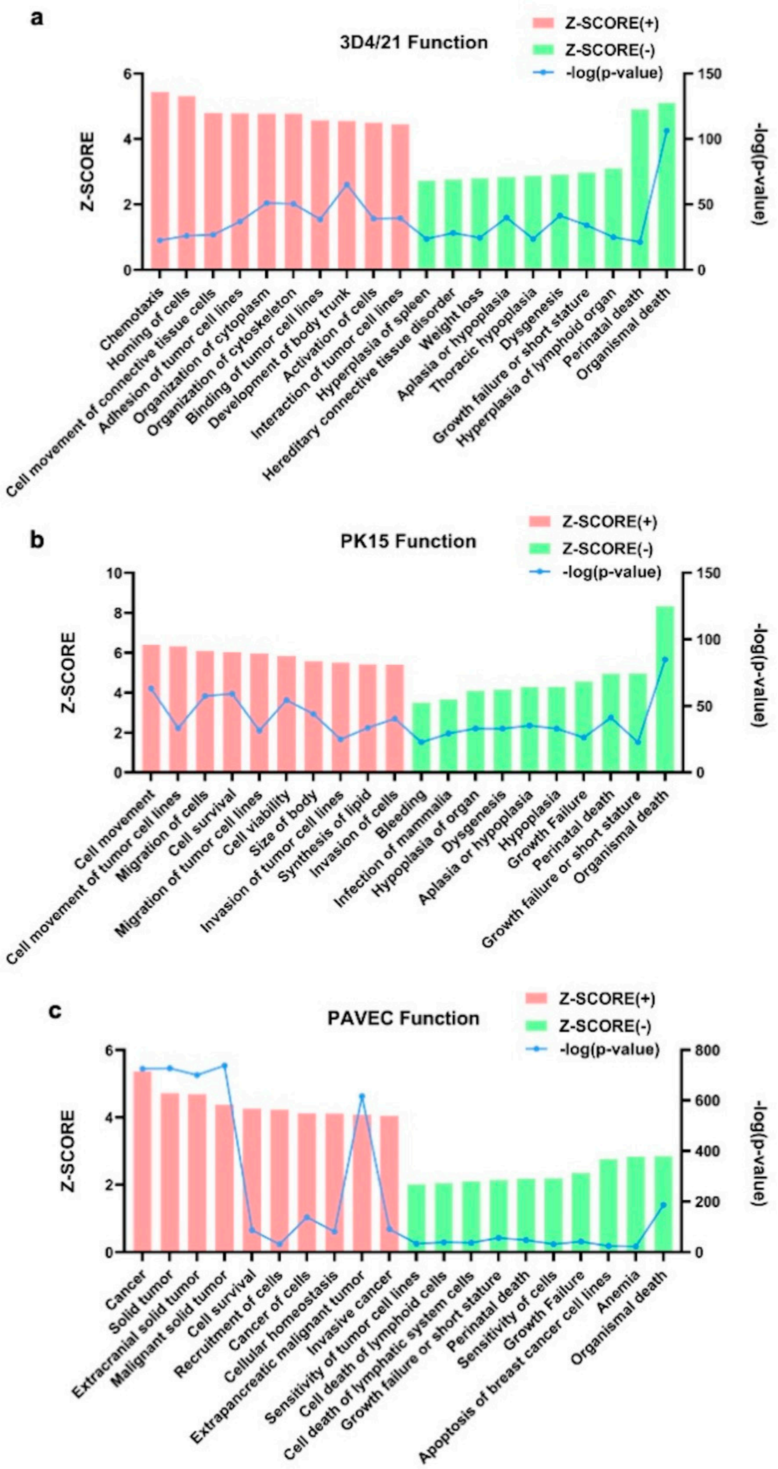


**Figure S4.** Top ten up-regulated and top ten down-regulated functions in three types of cells. Categories are shown in terms of the Z-SCORE, as represented by the left y-axis and the -log (p-value), represented by the right y-axis. (a) Top ten up-regulated and top ten down-regulated functions in the comparison of 3D4/21_*Gps* vs. 3D4/21_WT. (b) Top ten up-regulated and top ten down-regulated functions in the comparison of PK15_*Gps* vs. PK15_WT. (c) Top ten up-regulated and top ten down-regulated functions in the comparison of PAVEC _*Gps* vs. PAVEC _WT.

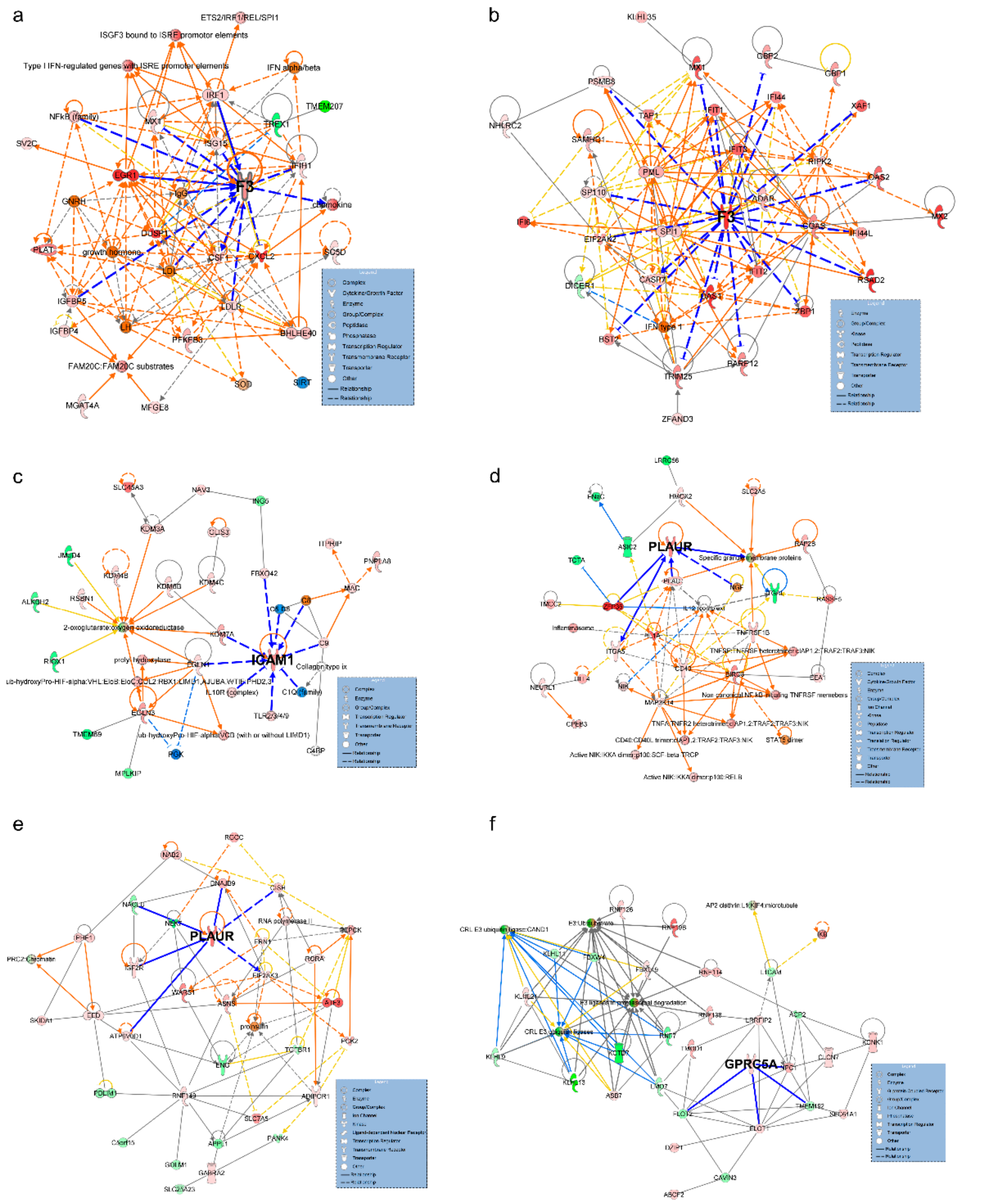


**Figure S5.** Networks for putative receptor candidates in different kinds of cells. (a) Gene network containing F3 in PK15_GPS vs. PK15_WT related to cellular movement, inflammatory response, skeletal and muscular system development and function. (b) Gene network containing F3 in PAVEC_GPS vs. PAVEC _WT related to antimicrobial response, immunological disease, inflammatory response. (c) Gene network containing ICAM1 in PK15_GPS vs. PK15_WT related to cancer, hematological disease, immunological disease. (d) Gene network containing PLAUR in PK15_GPS vs. PK15_WT related to cell-to-cell signaling and interaction, hematological system development and function, inflammatory response. (e) Gene network containing PLAUR in PAVEC_GPS vs. PAVEC _WT related to carbohydrate metabolism, cellular movement, connective tissue development and function. (f) Gene network containing GPRC5A in PAVEC_GPS vs. PAVEC _WT related to developmental disorder, hereditary disorder, metabolic disease.
